# Partially-supervised protein subclass discovery with simultaneous annotation of functional residues

**DOI:** 10.1186/1472-6807-9-68

**Published:** 2009-10-26

**Authors:** Benjamin Georgi, Jörg Schultz, Alexander Schliep

**Affiliations:** 1 Max Planck Institute for Molecular Genetics, Dept. of Computational Molecular Biology, Ihnestrasse 73, 14195 Berlin, Germany; 2 Biozentrum, Dept. of Bioinformatics, Universität Würzburg, 97074 Würzburg, Germany; 3 Current address: Dept. of Computer Science and BioMaPS Institute for Quantitative Biology, Rutgers, The State University of New Jersey, Piscataway, NJ, 08854, USA; 4 Current address: Department of Genetics, University of Pennsylvania, 528 CRB, 415 Curie Blvd PA 19104 Philadelphia, USA

## Abstract

**Background:**

The study of functional subfamilies of protein domain families and the identification of the residues which determine substrate specificity is an important question in the analysis of protein domains. One way to address this question is the use of clustering methods for protein sequence data and approaches to predict functional residues based on such clusterings. The locations of putative functional residues in known protein structures provide insights into how different substrate specificities are reflected on the protein structure level.

**Results:**

We have developed an extension of the *context-specific independence *mixture model clustering framework which allows for the integration of experimental data. As these are usually known only for a few proteins, our algorithm implements a partially-supervised learning approach. We discover domain subfamilies and predict functional residues for four protein domain families: phosphatases, pyridoxal dependent decarboxylases, WW and SH3 domains to demonstrate the usefulness of our approach.

**Conclusion:**

The partially-supervised clustering revealed biologically meaningful subfamilies even for highly heterogeneous domains and the predicted functional residues provide insights into the basis of the different substrate specificities.

## Background

Protein families frequently can be divided into subfamilies of similar but distinct function. The study of these subfamilies and the residues which control the functional specificity is an important step in the analysis of these families.

Many previous studies have focused on the question of how to find the functional residues for a given protein family when proteins already have been assigned to subfamilies. These methods include approaches based on information-theoretical measures such as relative entropy [1,2] or mutual information [3], template-based similarity scores to known functional residues [4], approaches which contrast position-specific conservation in orthologues and paralogues [5] or superfamilies [6] and comparisons to known reference 3D structures to find discriminatory surface residues [7]. The opposite of this so called *supervised *problem, is the *unsupervised *setup, where subfamily assignments are unknown and have to be inferred from the data. In the *unsupervised *case, clustering approaches for protein data can be applied to obtain subfamilies from set of protein sequences. For protein subfamily clustering most methods rely on the construction of a phylogenetic tree. These methods can be further subdivided into pure clustering methods [8-10] and approaches which include functional residue prediction [11-14]. Tree-based methods perform well in the presence of abundant data but might suffer from instability in the inferred topology for small data sets with strongly divergent sequences. For this type of data sets a stable phylogeny often cannot be inferred. In the worst case, a substrate specificity evolved multiple times independently leading to functional clusters which are not monophyletic. Here, any tree-based approach is assured to fail. Such data sets for instance arise from detailed molecular studies, where individual proteins are examined for their substrate specificity [15,16] and each experiment requires considerable effort. For such data sets a method, which is not based on a phylogenetic tree is advantageous. In a typical biological scenario, one is usually in between the *supervised *and *unsupervised *setting. A few members of a family have been characterized experimentally, but no functional information is known for the majority of the domain family. In these *partially-supervised *learning problems the classification error can be greatly reduced by making use of the annotated (labeled) proteins, even when only a small number of high quality labels is available [17]. In addition, the integration of a limited amount of expert knowledge can help to focus the clustering on subfamilies which are of relevance for a particular research question. In [18], we presented a method based on the *context-specific independence *(CSI) mixture framework for clustering of protein sequences and simultaneous prediction of functional residues.

The novel contribution of this work is as follows. First, the method has been extended and augmented with a *partially-supervised *learning setup, which allows the integration of prior knowledge into the clustering procedure. This extension allows the integration of experimental data for a few characterized proteins mimicking closely the typical biological scenario. Secondly, we simultaneously discover subfamilies and predict functional residues in four data sets of protein domain families. The results not only underline the relevance of our method but also allow insights into the sequence level basis of different ligand specificities. Thirdly, we contrast the clustering performance of our method with state-of-the-art tree-based approaches.

## Methods

### Mixture models

**Figure 1:**
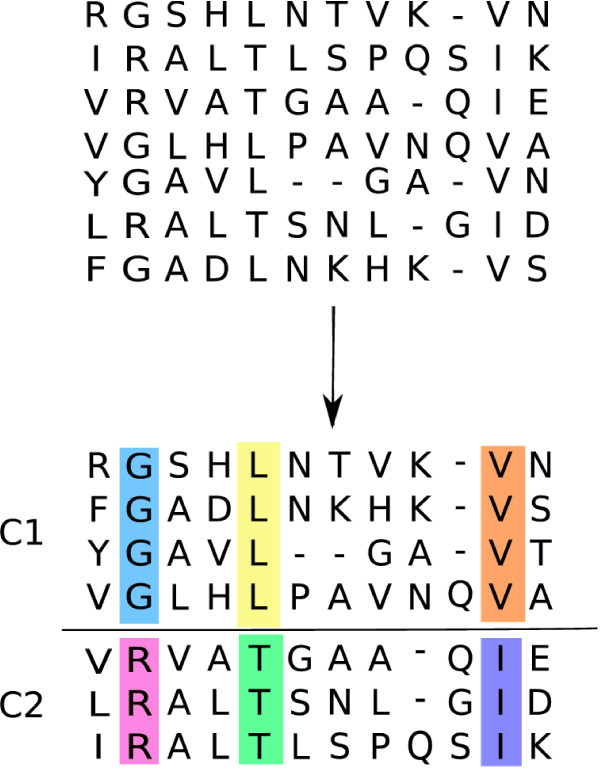
**Example method input and output**. **Top: **The input to the method is a MSA of protein sequences. **Bottom: **The output is a clustering into subfamilies (C1, C2) and annotation of putative functional residues (colored columns)

The general problem addressed in this work is visualized in Fig. 1. Given a multiple sequence alignment (MSA) of protein sequences (top), we discover subfamilies of sequences with different functional specificities (C1, C2) and simultaneously predict residue positions which are causal for these functional differences. This is indicated by the differently colored columns (bottom). Generally speaking, there is an increased subfamily-specific sequence conservation at positions which are relevant with respect to the distinct functions of the subfamilies. These positions are highly informative for the characterization of the clusters. Conversely, positions which are not relevant for the functional characterization of the subfamilies may show very little variability between subfamilies. Such positions are very weakly informative for the clustering and are best modeled by the same set of parameters in all subfamilies, instead of separate sets of parameters for each cluster. This notion of adaptation of model complexity to the position-specific degree of sequence variability observed in the data is formalized in the CSI mixture models, which are defined as follows:

Let *X*_1_, ..., *X*_*p *_be discrete random variables over the 20 amino acids and a gap symbol representing rows of a multiple sequence alignment (MSA) with *p *positions. For a given data set with *N *samples *D *= *x*_1_, ..., *x*_*N*_, a conventional mixture model is defined as

(1)

where *π *= (*π*_1_, ..., *π*_*K*_) are the non-negative mixture weights which sum to one, . That is, the data is modeled as a convex combination of *K *component distributions. As component distributions we employ a product distribution over the *p *features of the data set, the well known naive Bayes model [19],

(2)

The complete set of parameters of the mixture *M *is then given by Θ = (*π*, *θ*_1_, ...,*θ*_*k*_). This adoption of the naive Bayes model as component distributions implies the assumption of conditional independence between features given the mixture component. Moreover the standard assumption of independence between samples is made, which means that the probability of *D *under the mixture *P*(*D*|Θ) decomposes into the product over the probabilities of the individual samples,

(3)

**Figure 2:**
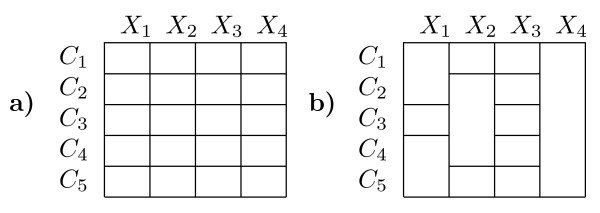
**Example mixture model structures**. **a) **Model structure for a conventional mixture with 5 components and four RVs. Each cell of the matrix represents a set of parameters *θ*_*kj *_in the mixture and every RV has an unique distribution in each component. **b) **CSI model structure. Multiple components may share the same set of parameters for a RV as indicated by the matrix cells spanning multiple rows. In example *C*_2_, *C*_3 _and *C*_4 _share the same set of parameters for *X*_2_.

The *context-specific independence *(CSI) extension to the conventional mixture framework is based on the observation, that in the latter, a separate set of parameters has to be estimated from the data for each component and each feature. This situation is visualized in the left matrix in Fig. 2. The example shows a five component mixture *C*_1_, ...,*C*_5 _over four features *X*_1_, ..., *X*_4_. Each cell in the matrix represents a separate set of parameters *θ*_*kj *_in the mixture. Now, the CSI framework is based on the insight that for many data sets it will be unnecessary to have a separate *θ*_*kj *_for all components in each feature. Rather, components should share parameters based on the feature-specific degree of variability observed in the data. An example of a CSI structure is shown on the right in Fig. 2. Again each cell of the matrix represents a set of parameters, and cells spanning multiple rows represent shared sets of parameters between components. For instance, components *C*_4 _and *C*_5 _share a parameterization for feature *X*_1 _and all components share parameters for feature *X*_4_.

In the following we introduce the CSI mixture model formally. Given the set of *K *component indexes *C *= {1, ..., *K*} and random variables (RVs) *X*_1_, ..., *X*_*p *_let *G *= {*g*_*j*_}_(*j *= 1,...,*p*) _be the CSI structure of the model *M*. Then *g*_*j *_= (*g*_*j*1_, ...*g*_*j*_*_Z_*_*j*_) where *Z*_*j *_gives the number of subgroups for *X*_*j *_and each *g*_*jr*_, *r *= 1, ..., *Z*_*j *_is a subset of component indexes from . Therefore, each *g*_*j *_is a partition of  into disjunct subsets, such that each *g*_*jr *_represents a subgroup of components with the same distribution for *X*_*j*_. The CSI mixture distribution is then obtained by replacing *f*_*kj*_(*x*_*ij*_|*θ*_*kj*_) with *f*_*kj*_(*x*_*ij*_|*θ*_*g_j_*(*k*)*j*_) in (2) where *g*_*j*_(*k*) = *r *such that *k *∈ *g*_*jr*_. Accordingly,  is the full model parameterization and  denotes the different parameter sets in the structure for feature *j*. The complete CSI model *M *is then given by *M *= (*G*, Θ).

Finally, the ranking of positions in the MSA for prediction of functional residues was carried out using the relative entropy score based on the CSI structure and the model parameters described in [18]. Essentially, this score contrasts the amino acid distribution within a subgroup with the distribution observed for all other subgroups. It can be seen as an extension of the score applied in [1] to the soft subgroup assignments inherent to the probabilistic mixture framework.

### Partially supervised learning

When clustering a given data set *D*, we need to infer the mixture parameters Θ and the CSI structure *G*. The standard technique for arriving at estimates for Θ is the *Expectation Maximization *(EM) algorithm [20]. The structure learning of *G *is carried out by applying the structural EM framework [21,22]. The central quantity of both of these algorithms is the posterior distribution of component membership

(4)

Here *τ*_*ki *_gives the probability that a sample *x*_*i *_was generated by component *k*. This posterior not only gives the solution to the conditional expectation of the hidden data (i.e. the assignment of samples to components) in the EM framework, it also gives rise to the expected sufficient statistics required for the structural EM algorithm (see [22] for details).

**Figure 3:**
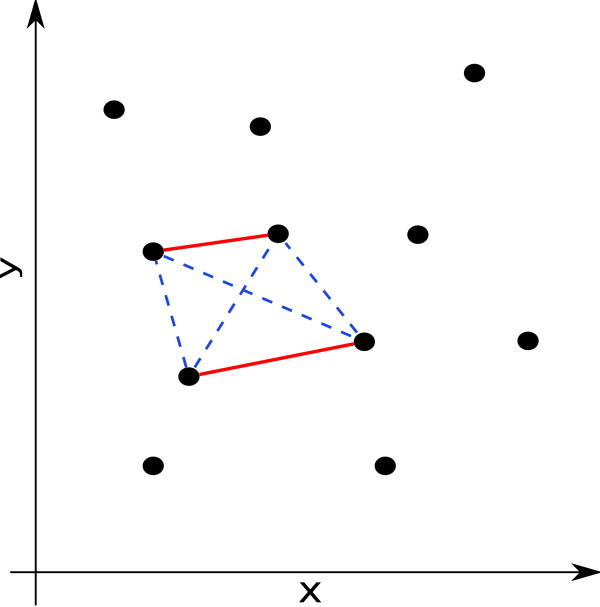
**Example of constraints arising from labeled samples**. Red edges between points indicate positive must-link constraints, blue edges negative *must-not-link *constraints.

A different interpretation of the component membership posterior is as the uncertainty in the cluster assignment of a sample. In fact, the entropy of this posterior can be used to identify samples where no clear classification is possible (e.g. [23]). In the partially-supervised setting, the component assignment of the labeled samples is known *a priori*. This means that for a labeled sample *x*_*i *_with label *l*, *τ*_*ki *_= 1 for *k *= *l *and *τ*_*ki *_= 0 for all *k *≠ *l*. Therefore, all samples with the same labels are constrained to be assigned to the same cluster, and therefore to different clusters if the labels are different. Fig. 3 shows an example for the constraints implicit in the labeling of data samples. Red edges between points represent *must-link *constraints, where each red edge stands for a different label, blue dashed edges *must-not-link *constraints. This setup can also be thought of as a point in the continuum between *supervised *(complete data) and *unsupervised *(incomplete data) learning tasks. For the former, the assignment of samples to components is known (i.e. the posterior takes the form given above). For the latter, the assignment of samples to components is unknown and the EM algorithm needs to be used to arrive at estimates for Θ. The partially supervised approach, then, is a situation, where there is complete data for a subset of samples in the data set. The same modification to the posterior for the labeled samples that defines the partially supervised EM, also gives the adaption of the CSI structure learning for the partially supervised setting.

### Structure learning

The structural EM algorithm allows the efficient evaluation of candidate CSI structures. In order to score the different structures we adopt a Bayesian approach and compute the model posterior given by



where *P*(*D*|*M*) is the probability of the data set *D *under the mixture *M *given by



where *P*(*D*|) is Eq. 3 evaluated for the *maximum a posteriori *parameters . *P*() is a Dirichlet mixture prior (DMP) based on nine basic chemical properties of the amino acids introduced in [18]. The prior *P*() regularizes the structure learning by introducing a notion of amino acid similarity into the objective function *P*(*M*|*D*). That is, a DMP *P*() is given by

(5)

where in this case *G *= 9 and each of the components represents one of nine basic chemical property of amino acids (e.g. hydrophobicity, size or charge). The parameters of the DMP component distributions *α*_*g *_and mixture weights *q*_*g *_are chosen according to the heuristic described in [18].

Finally, *P*(*M*) is a prior over the model structure given by the simple factored form

(6)

where *P*(*K*) = *γ*^*K *^is the prior over the number of components and  is the prior over model structure. *γ *< 1 and *α *< 1 are hyper parameters which determine the strength of the bias for a less complex model expressed in *P*(*M*). The values of *α *and *γ *were chosen by use of the heuristic introduced in [22] with a prior strength of *δ *= 0.05.

### Improvement of the structure learning algorithm

**Figure 4:**
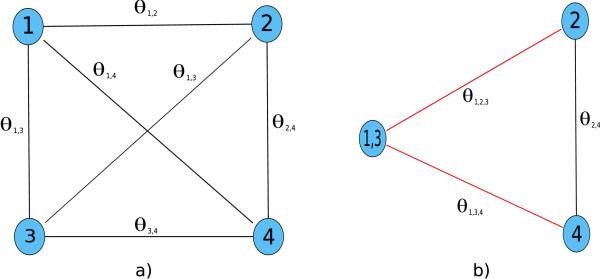
**Structure learning optimization strategy**. **a) **Pair-wise merges to be evaluated in the first step of the greedy structure learning for a four component mixture. **b) **Second step after *θ*_1,3 _has been accepted in a). Only the parameters corresponding to the red edges need to be recomputed.

The model posterior *P*(*M*|*D*) defines a scoring function over the space of CSI structures. However, exhaustive enumeration of all possible structures is infeasible in practice, since the number of possible structures is increasing exponentially with the number of mixture components. The number of possible structures for a given model can be computed by the Bell numbers [24]. For instance for a single feature and ten components there are already 115,975 possible structures to be evaluated. So, in order to arrive at structure estimates we applied an iterative, greedy procedure starting from the full structure matrix (see Fig. 2a for an example). Moreover, we optimized the computations by making use of redundant terms between the current model and the candidate models. In each step of the structure learning procedure, all pair-wise merges of groups in the current structure have to be scored and the one which maximizes the posterior is accepted. An example of this is shown in Fig. 4a). Each of the four nodes represents a component of the mixture and each pair of components gives rise to a merge parameter *θ*_*gjr *_based on the expected sufficient statistics of the merge, which in turn allows the evaluation of the model posterior *P*(*M*|*D*). This means that in each step O() candidate merges have to be computed, where *Z*_*j *_is the current number of groups, starting with *Z*_*j *_= *K *in the first step. An important observation that can be made, is that the merge parameters *θ*_*gjr *_of disjunct merges are independent in the sense that the respective computations have no terms in common. This is because the merge parameters are computed from the element-wise addition of the component membership posteriors *τ*_*k *_= {*τ*_*ki*_}_*i *= 1,...,*N *_of the components that are part of the merge. An example is *θ*_1,3 _and *θ*_2,4 _in Fig. 4a). The former is based on *τ*_1,3 _= *τ*_1 _+ *τ*_3_, whereas the latter arises from *τ*_2,4 _= *τ*_2 _+ *τ*_4_. If we were to accept the merge of 1 and 3 in the first step, the second step (shown in Fig. 4b)) would necessitate the re-computation of only the merge parameters *θ*_1,2,3 _and *θ*_1,3,4 _(edges shown in red), whereas *θ*_2,4 _would remain unchanged from the previous step and need not be computed again. Therefore, by caching the merge parameters in each step, the number of merge parameters to be re-evaluated in each step after the first drops from *O*() to *O*(*Z*_*j*_). This greatly increases the speed of the structure learning, especially for models with a large number of components.

## Results

**Figure 5:**
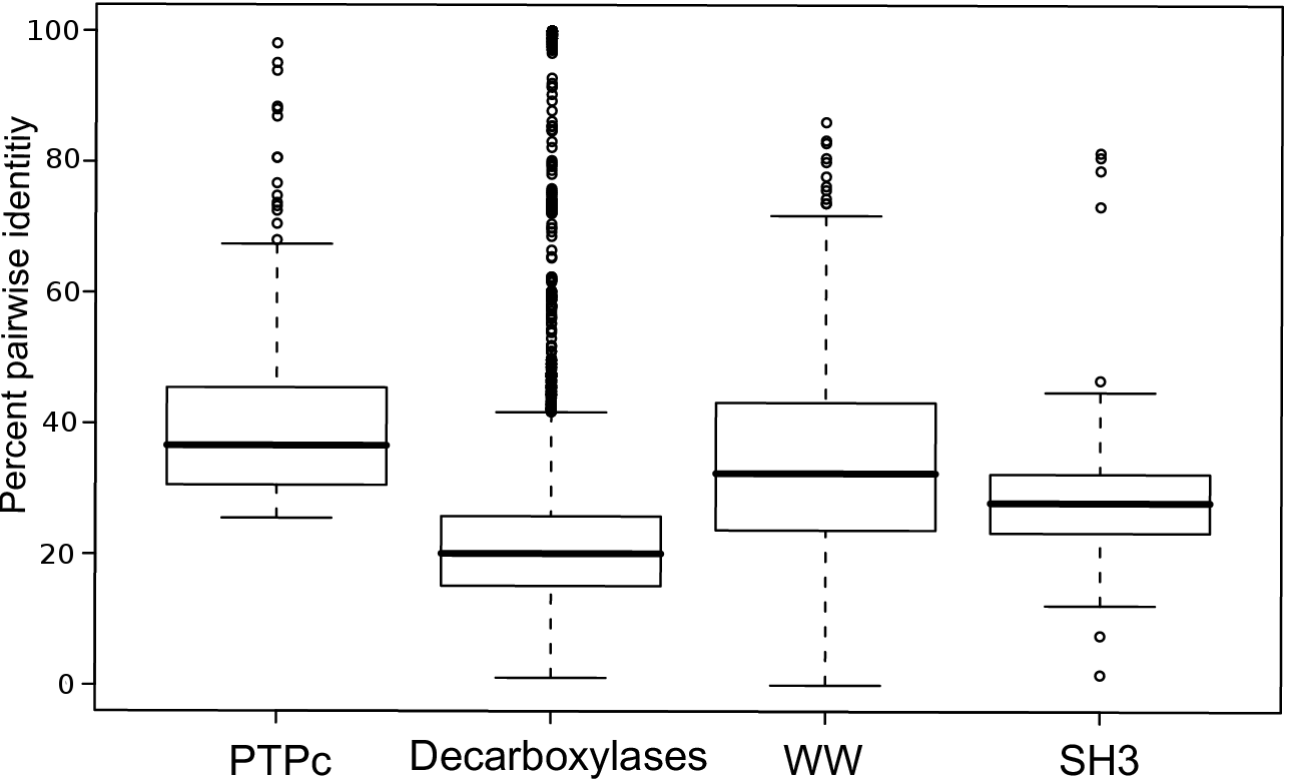
**Global pair-wise sequence identity for the four data sets**. Large parts of each data set lie within the twilight zone of ≤ 35% sequence identity.

We applied our mixture modeling to four protein sequence data sets. These were chosen as representatives of specific challenges for subclass discovery. In the case of the phosphatases, catalytic active members should be distinguished from inactive ones. The focus in this example is the automatic identification of sites important for the catalytic reaction. The second dataset, pyridoxal-5'-phosphate dependent decarboxylases was chosen to test whether our algorithm is able to identify substrate specific groups even if they were not monophyletic. In this case, one substrate specificity was evolved independently in archaea and plants. Thus, no tree based approach will correctly predict the two classes of substrate specificity. Furthermore, this protein family is highly divergent, with median pairwise identities below 20%. This also holds true for the remaining two datasets, WW and SH3 domains (Fig. 5). Additionally, these domains are comparably small, leaving few conserved positions for classification. Finally, they are functionally divergent and genetically mobile, that is they can be found in many otherwise non-homologous proteins. Finally, as part of the comparison of clustering performance and in order to assess the impact of the clustering quality on the functional residue prediction, we also revisit a data set of malate/lactate dehydrogenase sequences previously analyzed in [18]. All the alignments are available from the authors upon request. The general properties of all data sets are summarized in Table 1.

**Table 1:** General properties of the data sets.

**Data**	**Size**	**length**	**length after filtering**
phosphatase	22	316	259
decarboxylase	72	411	304
WW	49	40	36
SH3	20	81	56
dehydrogenase	29	168	141

The number of sequences, length of alignment and length of alignment after filtering (see below) are given.

In order to assess the impact of the partially-supervised setup, models were trained with and without labels. The labeled samples were chosen at random. For each data set we trained models with a number of components in a range of one to ten and the *Normalized Entropy criterion *(NEC) [25] was used to perform model selection.

In practice, it seems reasonable to expect that the number of labeled data points available for a given data set is fairly limited. Therefore, the main question we were interested in with regards to the partially-supervised learning was in how much different amounts of randomly chosen labels would impact on the clustering performance.

Following the approach in [18], alignment columns with more than 33% gaps were filtered prior to clustering in order to reduce the noise level in the data sets. The clustering quality was evaluated by the standard measure of accuracy , where for the cluster labels and the true class labels, *TP *gives the number of pairs of samples where the true class labels and the cluster labels are the same. The remaining quantities are calculated accordingly, i.e. *FP *(same cluster, different class), *TN *(different cluster and class) and *FN *(different cluster, same class).

### Receptor tyrosine phosphatases

Together with protein kinases, protein phosphatases are the key players of signal transduction cascades. Here, we analyzed a specific subfamily, the receptor tyrosine phosphatases. Intracellularly, receptor tyrosine phosphatases contain two phosphatase domains. Whereas the membrane proximal domain is catalytic active, the distal domain has lost its activity and is assumed to be involved in regulation. Searching for differences in site specific evolutionary rates, a second functional region, opposing the catalytic center, was proposed [26]. Using this dataset, we asked two questions: (i) is our algorithm capable to recover a classification which is based on phylogenetic trees [27] and (ii) can the algorithm identify known functional sites.

Therefore, the 22 phospho-tyrosine phosphatase domains (PTPc) of all proteins with two PTPc domains in the human genome were extracted from *SMART *[28]. An alignment was obtained using *MUSCLE *[29].

When performing the clustering, a clear separation of the two classes became apparent. The NEC model selection chose two clusters as optimal and the resulting model separated the classes with perfect accuracy. This result was confirmed in 30 repetitions and unsurprisingly remained unchanged when adding prior knowledge in form of labeled samples.

**Figure 6:**
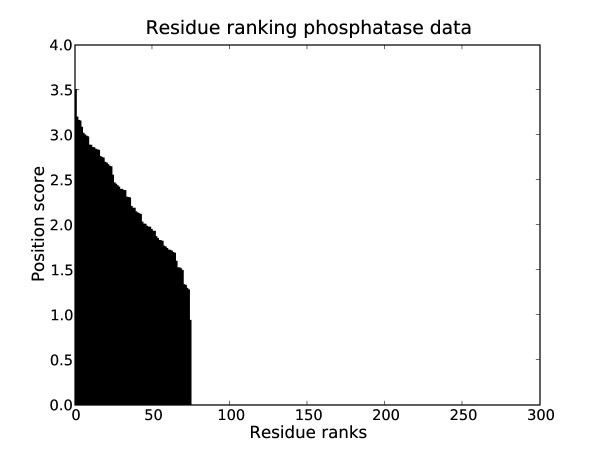
**Ranking of alignment positions for the phosphatase data set**. Ranking of positions in the alignment for the phosphatase data set.

**Figure 7:**
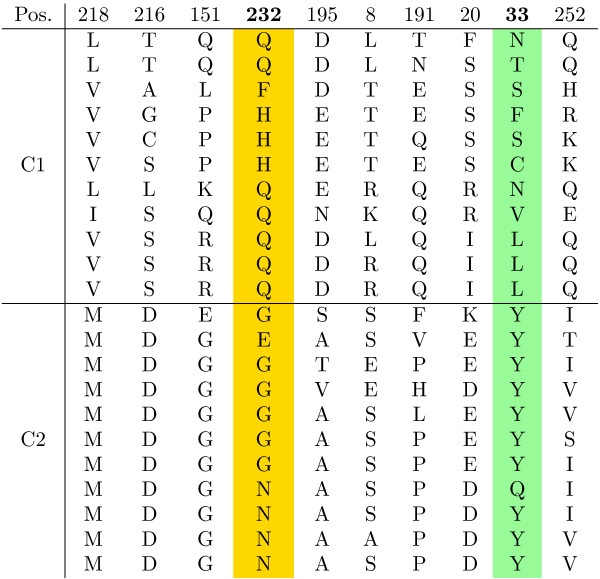
**Best ranking positions for the phosphatase data set**. Top ten ranked alignment positions on the phosphatase data set. The two experimentally found sites 232 and 33 are highlighted in yellow and green.

Fig. 6 shows the ranking of alignment positions by score. Positions with a score of zero have been marked as uninformative for the clustering by the CSI structure learning. This is the case for the majority of positions. When considering the top ranked positions, we found two positions (Gln232 and Leu33 with respect to sequence [PDB:1LAR]) which are part of a loop surrounding the active site (Gln232) or involved in inter domain hydrogen binding (Leu33). To illustrate the kind of regularities in the alignment revealed by the position ranking, the top ten ranked positions in the phosphatase data are shown in Fig. 7. The two experimentally confirmed sites are highlighted in yellow and green, respectively. Indeed, the method picked up positions which showed patterns of subgroup-specific conservation.

### Pyridoxal-dependent decarboxylase

The pyridoxal-5'-phosphate dependent amino acid decarboxylases comprise a large protein family of four evolutionary unrelated families [30]. These are classified according to their substrates. Here, we focused on two enzymatic classes of group II decarboxylases. The first catalyses the decarboxylation of tyrosine (EC: 4.1.1.25), whereas glutamate is the substrate of the second group (EC: 4.1.1.15, identifier starting with DCE). The data set was constructed by selecting all sequences of the PFAM family *pyridoxal_deC *which had an unique annotation for the substrate specificity in the **CATALYTIC ACTIVITY **field of the corresponding SWISSPROT entries. This resulted in 35 sequences with glutamate specificity and 37 sequences with specificity for tyrosine. An alignment of these 72 sequences was obtained using *Clustalw *[31]. Subsequently, a phylogenetic tree was calculated using *proml *[32], which showed that the studied tyrosine decarboxylases are not monophyletic. Whereas the position of the sequences within the groups were frequently only poorly supported, the relation of the four groups to each other was revealed in all of the 100 bootstraps [see Additional file 1: Supplemental Figure S1]. Thus, the catalytic specificity for tyrosine arose independently in archaea (identifier starting with MFNA) and plants (TYDC). No tree based method will be able to identify the two groups with the differing substrate specificity. We used this dataset to test if our method was able to dig out the small signal of substrate binding differences covered by a strong phylogenetic signal.

**Figure 8:**
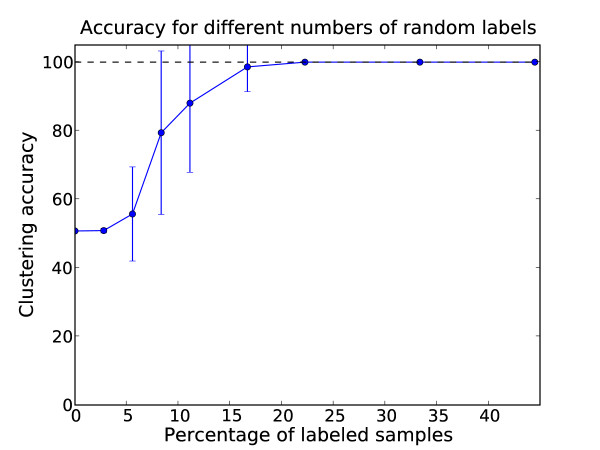
**Evaluation of accuracy for different amounts of labels on the decarboxylase data**. Average accuracy for different numbers of randomly chosen labels for the pyridoxal-dependent decarboxylase domain data set. The error bars show the standard deviation over thirty repetitions.

While the NEC assigned two clusters, the separation of the glutamate and tyrosine subclasses proved to be very challenging without labels. The average performance over 30 repetitions was an accuracy of 51% (SD: 0.3%). When adding the power of the partially supervised framework to the clustering by randomly selecting different numbers of labels for the two subclasses a different picture emerged. The average accuracies based on 30 repetitions for different amounts of randomly selected labels per class are shown in Fig. 8. The average clustering accuracy increased monotonously with the number of labeled samples. While the average accuracy improved significantly in the range of 5%-16% labeled samples, the variance also increased. This is most likely an effect of the random selection of labels. Indeed, the quality of the labeling is crucial for the performance of partially-supervised approaches [33]. When outliers of a class were labeled, the labeling could even have a negative impact on the clustering performance. This issue is increasingly receiving attention in the machine learning community [34]. For about 22% labels the variance decreased again and models with perfect accuracy and zero variance (over the 30 repetitions) were obtained for more than 22% labels.

The unsupervised clusterings returned a very low-variance, highly robust clustering of the sequences. These clusterings did not reflect the tyrosine/glutamate subfamilies and so the question was whether they represent some other biological context. Upon examination it became clear that the unsupervised clustering split the data set based on phylogenetic divergence. One cluster contained predominantly archaeal and bacterial sequences, the other metazoa and viridiplantae (i.e. green plants). Based on this taxonomic classification of the sequences, the clusterings had an average accuracy of about 82%. This means that for the unsupervised setup, the clustering picked up a decomposition of the sequences which, while being biologically meaningful in itself, did not reflect the specific question we were interested in. This problem was overcome by including prior knowledge in form of sequence labels. These results illustrated nicely how the partially supervised approach improved the parameter estimation and structure learning by guiding it away from clusterings which were not consistent with the biological question under consideration. However, the high variance for moderate amounts of labeled samples again underlined the importance of the label selection procedure.

**Figure 9:**
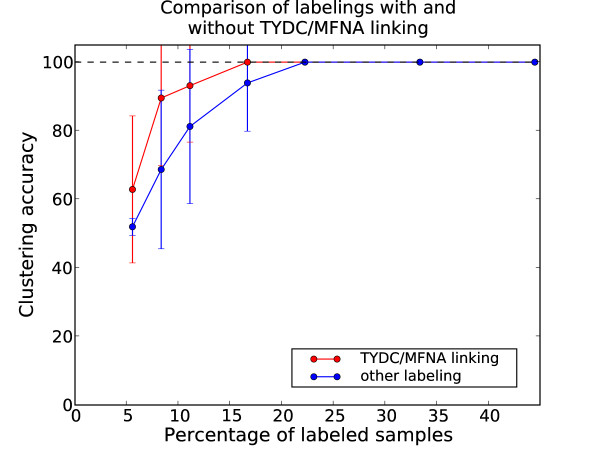
**Evaluation of accuracy for different amounts of labels linking TYDC and MFNA**. Average accuracy for different numbers of randomly chosen labels for cases where the labeling links TYDC and MFNA sequences (red) and other labelings (blue). The standard deviation over thirty repetitions is shown by the error bars.

In order to quantify the impact of label selection, we split the results shown in Fig. 8 in cases where the phylogenetically divergent groups with glutamate specificity TYDC/MFNA were linked by the labeling (i.e. at least one TYDC and MFNA sequence was labeled) and cases where there was no link. The average accuracies for the two cases are shown in Fig. 9. Accuracies of the cases where TYDC/MFNA were linked are shown in red, other cases in blue. The average accuracies of labelings with TYDC/MFNA were higher. For 6 labels (~8% labels out of 72 sequences), the average accuracy improved from 69% to 90% for the cases where TYDC and MFNA were linked. It is also noteworthy that the variances of the average accuracies were still large. These results showed how the integration of additional biological prior knowledge (in this case the common function of TYDC and MFNA) could improve the clustering.

### WW domain family

The WW domain is a small protein module of about 40 amino acids, which forms a triple stranded anti-parallel *β*-sheet. It is involved in protein-protein interactions and its target sequences are usually proline rich or contain phosphorylated serine/threonine sites. Based on the sequence of their ligands, WW domains have been grouped into three functionally different classes (I, II/III and IV) [35]. Using protein arrays, the ligand preferences for 49 human WW domains of the two most populated classes, I and II/III were determined [15]. Here, we asked the question, whether the ligand based classification can be recovered using only the domain sequences. The NEC model selection gave a two cluster model as optimal. The domains were correctly classified with accuracy 96% with this model. This result was highly robust, 30 repetitions of the clustering gave an average accuracy of 96% (SD 1%). When performing the same setup in a partially-supervised manner with randomly selected labels, the same results were obtained for any number of labels.

**Figure 10:**
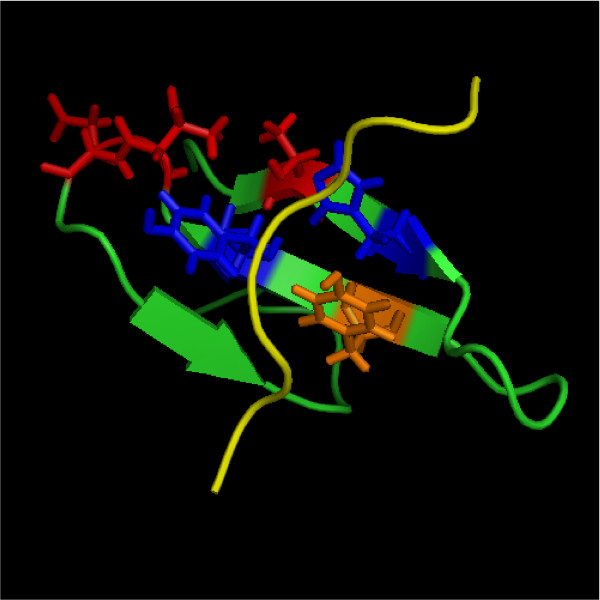
**Evaluation of predicted functional sites in the FBP11 WW1 structure**. Structure of FBP11 WW1. Highlighted are positions specific for ligand binding by the XP2 groove identified by our method (blue), not identified by our method (orange) and further positions identified as important for the classification (red). Ligand in yellow

**Figure 11:**
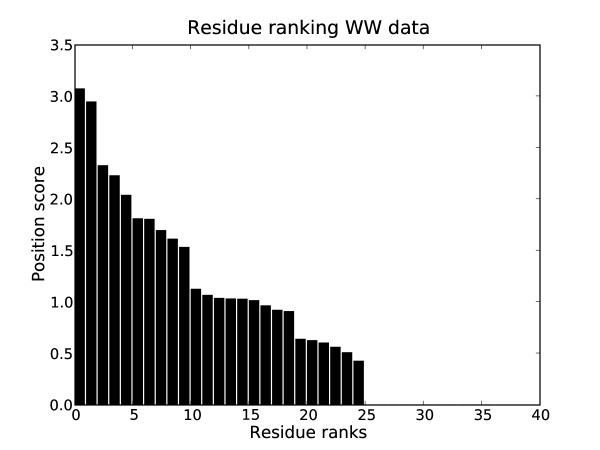
**Ranking of alignment positions for the WW data set**. Ranking of positions in the alignment for the WW data set.

Based on the optimal unsupervised clustering according to the NEC, the positions of the alignment were ranked by their information for separating the clusters (Fig. 11 shows the ranked scores). The main structural difference between members of Group I and of Group II/III is the existence of an additional binding site, the XP2 groove in the latter. Of three residues which form the XP2 groove [36], two were identified within the five top ranking positions (Fig. 10). Thus, our method was not only able to identify the correct clustering of this domain, it also identified the sites responsible for the functional difference without prior knowledge.

### SH3 domain family

Similar to the WW domain, the src homology domain 3 (SH3) is a protein interaction module binding to polyproline regions. Its preferred binding partners are characterized by a structural motive, the polyproline type II helix. Two types of binding mode can be distinguished based on the direction of the helix in the binding groove [37]. In contrast to the WW domain, these different binding preferences are not caused by two different binding patches on the domain, rather it is one site responsible for these interactions. This makes an automated classification and identification of specificity inducing sites even more challenging than in the case of the WW domain.

**Figure 12:**
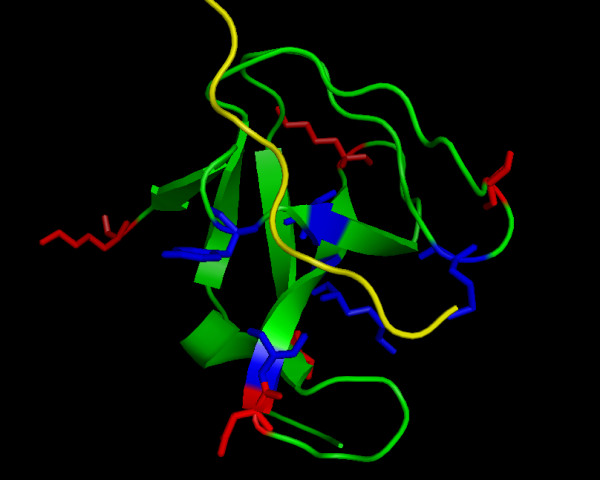
**Evaluation of predicted functional sites in the PEX13 SH3 domain structure**. Structure of Yeast SH3 domain from PEX13 (green) and bound peptide ligand (yellow). Residues of the canonical binding sites identified by our method in blue, further important sites in red

We analyzed data from a large scale interaction study on 20 yeast SH3 domains [16] which classified each domain into one of three groups (I, II and Unusual) based solely on their ligands. Some of the domains showed interactions with both types of polyproline helices, indicating that even biologically there is no clear separation between these groups. Out of the twenty SH3 domains in the data set, eight fell unequivocally into class I, four into class II, five showed binding for both classes (I and II) and three showed an unique, unusual binding pattern. For the partially-supervised clusterings 1-6 labeled samples from each of the classes I and II were selected at random. This corresponds to 10%-60% labeled samples out of the 20 sequences in the data set. The structure of the SH3 domain of PEX13 (Fig. 12) was used as reference.

**Figure 13:**
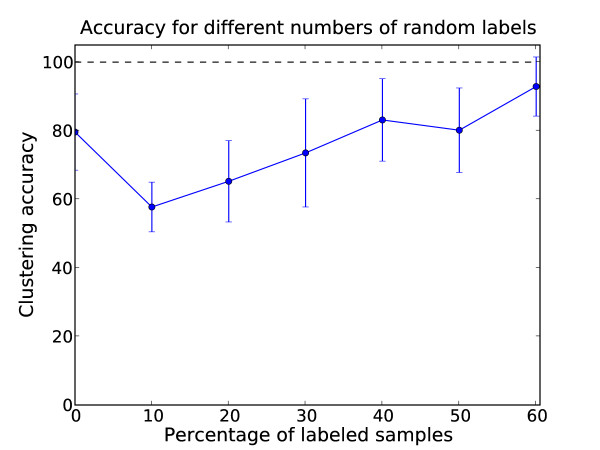
**Evaluation of accuracy for different amounts of labels on the SH3 domain data**. Average accuracy for different numbers of randomly chosen labels for the SH3 domain data set. The error bars show the standard deviation over thirty repetitions.

For the the models trained with two labels per class, NEC model selection picked two components as optimal and the clustering gave a clear separation of class I (with two of the unusual domains) and classes II and I/II (and the remaining unusual domain). The separation was complete except for two misclassifications, one for classes I and II each. With respect to the I vs. (II, I/II) class separation, this amounted to an accuracy of 78%. To quantify the robustness of this result and the performance for larger number of labels, average accuracies over thirty repetitions were computed Fig. 13. Thirty repetitions of the two label setup had an average accuracy of around 65% (SD 12%). In the unsupervised approach we observed average accuracies of 80% (SD 11%) for 30 repetitions. Thus, the partially-supervised setup had a detrimental effect on clustering performance with up to four labels per class. Probably the main reason was again the random selection of labels for the partially supervised parameter training. Although the average accuracy was better, the optimal unsupervised model chosen by NEC had one misclassification more than the one from the partially-supervised clusterings with two labels per class (three misclassifications instead of two).

**Figure 14:**
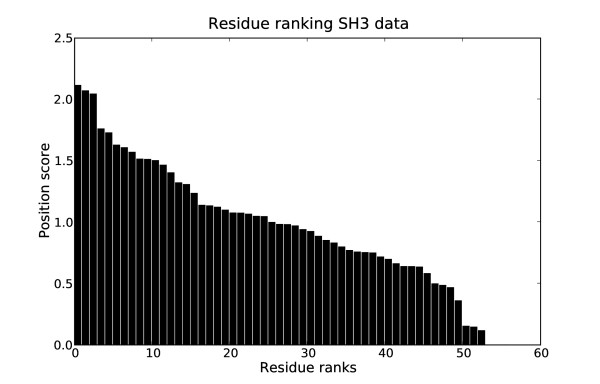
**Ranking of alignment positions for the SH3 data set**. Ranking of positions in the alignment for the SH3 data set.

**Figure 15:**
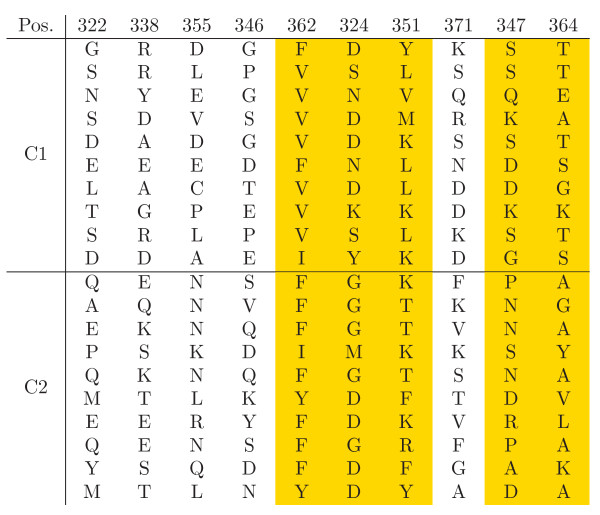
**Evaluation of predicted functional residues for the SH3 data**. Top ten ranked alignment positions on the SH3 data set. The experimentally verified ligand interacting sites are marked in yellow.

As the clustering results revealed a sufficient overlap with the ligand based classification, the next question was, whether the sites of highest importance for the clustering are indeed involved in the ligand binding. One interesting aspect of the CSI structure learned for the SH3 data was, that there were almost no completely uninformative positions in the alignment (Fig. 14). This showed the high heterogeneity of the sequences involved, where each position carried at least some information about cluster separation. In order to evaluate the ranking, the highest scoring sites were mapped on the structure of the Pex13p SH3 domain. This domain contains a second interaction site, which we did not consider, as it is not present in other members of this group. We found that of the 10 highest scoring residues 5 are directly involved in ligand interaction, i.e. "their accessible surface area dropped by more than 50% or their backbone amides were shifted considerably after binding of the ligand" [38] (Fig. 12). The alignment of the ten best scoring positions is shown in Fig. 15. The previously reported functional residues are marked in yellow.

Interestingly, other sites directly involved in the interaction yielded low scores. The SH3 domain of Sla1 ([PDB:2JT4]) contains five primary binding interface positions, Tyr362, Phe364, Trp391, Pro406 and Phe409. These positions were listed at the very end of the ranking, within the last nine positions, and were found to be uninformative for the clustering. Thus, our method identified those sites, which are responsible for differences in binding between the family members. Complementary, it rejected those, which are of importance in all family members.

### Comparison with other methods

The general principle underlying most methods for the prediction of functional residues, independent of the specific metric used, is to contrast the amino acid composition in the alignment between different clusters. As such, the quality of the clustering is crucial for the subsequent prediction of functional residues. Therefore, in the following we evaluate the clustering performance of the CSI mixtures.

In order to assess the performance of our method in comparison to other state-of-the-art procedures, we computed clusterings for all the data sets using five different methods. The *CLUSS *3.0 [10,39] algorithm is a tree-based clustering method which does not rely on an alignment. In the original papers CLUSS has been compared with favorable results to a variety of protein clustering methods. The clusterings were performed using the *CLUSS *server at . Additionally we applied four methods which combine clustering and functional residue prediction. The *ProteinKeys *algorithm [13] finds subgroups and putative functional residues by combinatorial entropy optimization . The *TreeDet *[14] server  offers two different methods for clustering and functional residue prediction: the *TreeDet-S *is a tree-based method which tries to find optimal tree cuts and *TreeDet-S3DET *is based on dimensionality reduction by multiple correspondence analysis. All methods were run on the respective servers with default parameters.

In addition to domains described in detail above, we revisited a previously analyzed data set [18]. The alignment of the malate/lactate dehydrogenase (LDH/MDH) family (PFAM PF00056) is a benign test case for clustering and functional residue prediction. The data set is small (29 sequences, 16 LDH, 13 MDH) and there is a single experimentally verified specificity-determining position (Arg86 with respect to PDB 1IB68) with very strong (almost perfect) subgroup specific conservation. For details on the CSI mixture clustering on this data, refer to [18].

Table 2 summarizes the accuracy values of all clusterings in comparison to the optimal unsupervised (i.e. unlabeled) results determined by the NEC model selection of the CSI mixtures. The WW data set was rejected by the *TreeDet *server for being too short (fewer than 50 amino acids).

**Table 2:** Method comparison. Comparison of accuracies of the CSI mixtures and the CLUSS, ProteinKeys, TreeDet-S and TreeDet-S3DET methods.

	**CSI mixtures**	**CLUSS**	**ProteinKeys**	**TreeDet-S**	**TreeDet-S3DET**
dehydrogenases	**100**%	76%	71%	81%	97%
SH3	**80**%	63%	61%	69%	57%
WW	**96**%	69%	64%	-	-
phosphatases	**100**%	85%	69%	64%	87%
decarboxylases	51%	68%	66%	49%	**77%**

Generally the CSI mixtures performed favorably. In the dehydrogenase data, there is a strong correlation between the clustering accuracy and the presence of the true specificity inducing residue in the predicted functional residues. For those methods with good clustering performance (CSI mixtures, TreeDet-S, TreeDet-S3DET) Arg86 was correctly predicted to be functional, whereas the other method (ProteinKeys) did not (note that CLUSS is a pure clustering method and does not generate predictions for functional residues). This underlined the importance of a high quality clustering as a necessary condition for the prediction of biologically valid functional residues.

The ProteinKeys method achieved rather mediocre clustering accuracies. The main reason was the inflated number of clusters returned by the method. This suggests that the ProteinKeys method has the implicit assumption that the data contains a larger number of functional subgroups, which makes it less suited for the small, highly diverse data sets which are the focus of this work.

To test whether this result was exemplary for other tree based methods, we calculated phylogenetic trees for the four analyzed domain families. Starting with the multiple alignments of the families, 100 bootstrap replicates were generated with the *seqboot *program [32]. Finally, trees were calculated using *proml *and a consensus tree was generated by *consense *[32]. The decarboxylase tree captured the phylogenetic partitioning as described above. As expected for the phosphatases, the inactive and the active members were separated with a bootstrap support of 100. This was in contrast with the other two data sets where the resulting tree did not show reliable bootstrap values [see Additional file 1: Supplemental Figure S2 - S4 for the bootstrap trees]. The average bootstrap values per data set were 41.2 (SD 34.7) for the SH3 data and 31.6 (SD 25.1) for the WW domains. From the high standard deviation it can be seen, that beside some edges with decent support, there were also edges with almost no support in the bootstrap samples. With the usually accepted bootstrap cutoff of 75, the usefulness of the tree was highly dubious. In contrast, our method gave reliable results even in the challenging cases of short and extremely divergent domains (Fig.5), which were intractable for tree based methods. For all four data sets the median identity was at or below (~35%) the so called protein twilight zone.

## Discussion & Conclusion

Domains are the structural, evolutionary and functional building blocks of proteins. A domain based analysis is a fundamental step in predicting the function of a protein. Still, this step is hampered by the fact that different members of the same domain family can perform vastly differing functions. Here, we used a CSI mixture clustering method augmented with partially-supervised learning to assign domains to a functional subclasses. Additionally, our method predicts residues relevant for the specific function of a subclass. This information can directly be used in the experimental characterization of the protein. The application of our method to four domain families revealed a good classification and identified known functional residues even for the selected, highly challenging families (receptor tyrosine phosphatases, decarboxylases, WW and SH3 domains).

It is interesting to contrast the different effects of the extension to partially-supervised learning on these four data sets. For the WW and phosphatase domain data, the unsupervised clustering already yielded a very high quality clustering and as such it is not unsurprising that a minimal number of labels did not have a significant effect on the results.

For the yeast SH3 domain data set, the addition of random labels actually caused more noise in the clustering than compared to the unsupervised case. There are a number of possible explanations for the apparent disadvantage the partially-supervised framework had on the SH3 data. First of all, the labels used for the 30 repetitions of the partially-supervised setup were chosen at random and therefore it is likely that samples which are outliers within their class were labeled. This can overemphasize these outliers in the characterization of the cluster center, especially if only few labels are available, as was the case in our setup. This effect of low quality labels having a detrimental effect on clustering performance has been described previously [33]. From this perspective, the results on the SH3 data can be seen as a cautionary tale to take proper care that the chosen labels chosen are of high quality. It is also noteworthy that even though the average performance of the partially-supervised method decreased, the best model chosen according to NEC was one misclassification better than the model obtained from the unsupervised setup. Finally, for the decarboxylase data the integration of prior knowledge greatly increased the quality of the clustering solution with respect to a subgrouping of the data that was informative for the biological classification we were studying. At the same time, just as with the SH3 data, the high variance observed for the labeled data sets underlined the importance of the choice of labels. Bearing that in mind, if the labeled samples are known to be of high quality, the partially-supervised approach can greatly improve the clustering setup.

In addition to the functional grouping, our method also provides a ranking of sites important for each group. As exemplified by the SH3 domain, the method is able to distinguish 'core' position, which are functional in all family members, from those which are responsible for the sub-group specific function. This information could guide the further experimental characterization of the domain family.

The software we developed to carry out this analysis *PyMix - the Python Mixture Package *is freely available from . For future work it would be interesting to investigate additional data sets, especially protein sequences which arose from the kind of high-detail studies which yield data sets that do not lend themselves to study with tree-based methods. On the theoretical side it would be interesting to extend the framework with more complex notions of partial-supervision such as pair-wise positive and negative constraints between samples. Finally, we intend to devise additional improvements to the running time of the structure learning to optimize performance for the analysis of large scale data sets.

## Authors' contributions

BG implemented the method and carried out the computations. JS assembled the data sets and performed the biological analyses. AS conceived the study and participated in its design and coordination. All authors wrote the manuscript and approved the final version.

## Supplementary Material

Additional file 1 **Supplementary figures**. Figures of bootstrap trees for the various data sets. Click here for file
