# Supplementary material for: Partially-supervised protein subclass discovery with simultaneous annotation of functional residues
Source: BMC Struct Biol. 2009 Oct 26;9:68. doi: 10.1186/1472-6807-9-68 (PMC2777906; doi:10.1186/1472-6807-9-68)
Supplement: Additional file 1 — Supplementary figures. Figures of bootstrap trees for the various data sets. [file 1472-6807-9-68-S1.PDF]

Supplementary Material for "Partially-supervised protein subclass discovery with simultaneous annotation of functional residues"

October 19, 2009

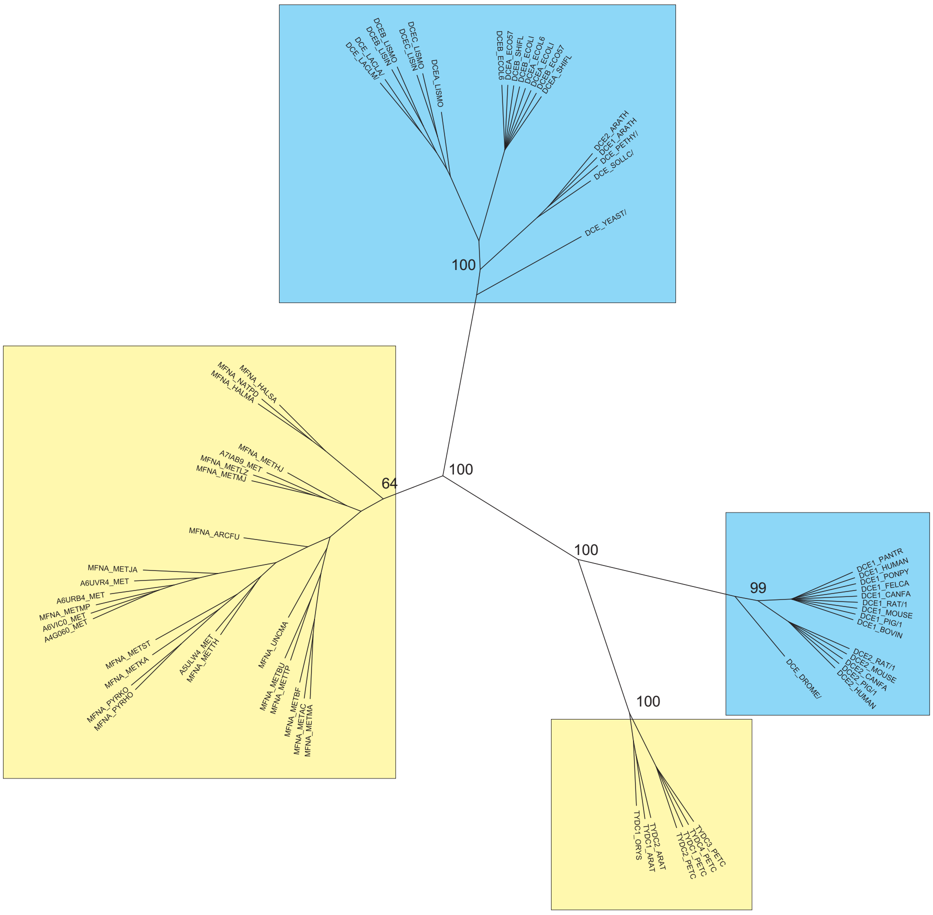

Figure 1: Phylogenetic tree of the analysed pyridoxal-dependent decarboxylases. Glutamate decarboxylases (EC: 4.1.1.15) are highlighted in blue, tyrosine decarboxylases (EC: 4.1.1.25) in yellow. Bootstrap support for the main branches is shown. MFNA_PYRFU and MFNA_PYRAB were excluded from the tree as they did not cover the whole decarboxylase domain.

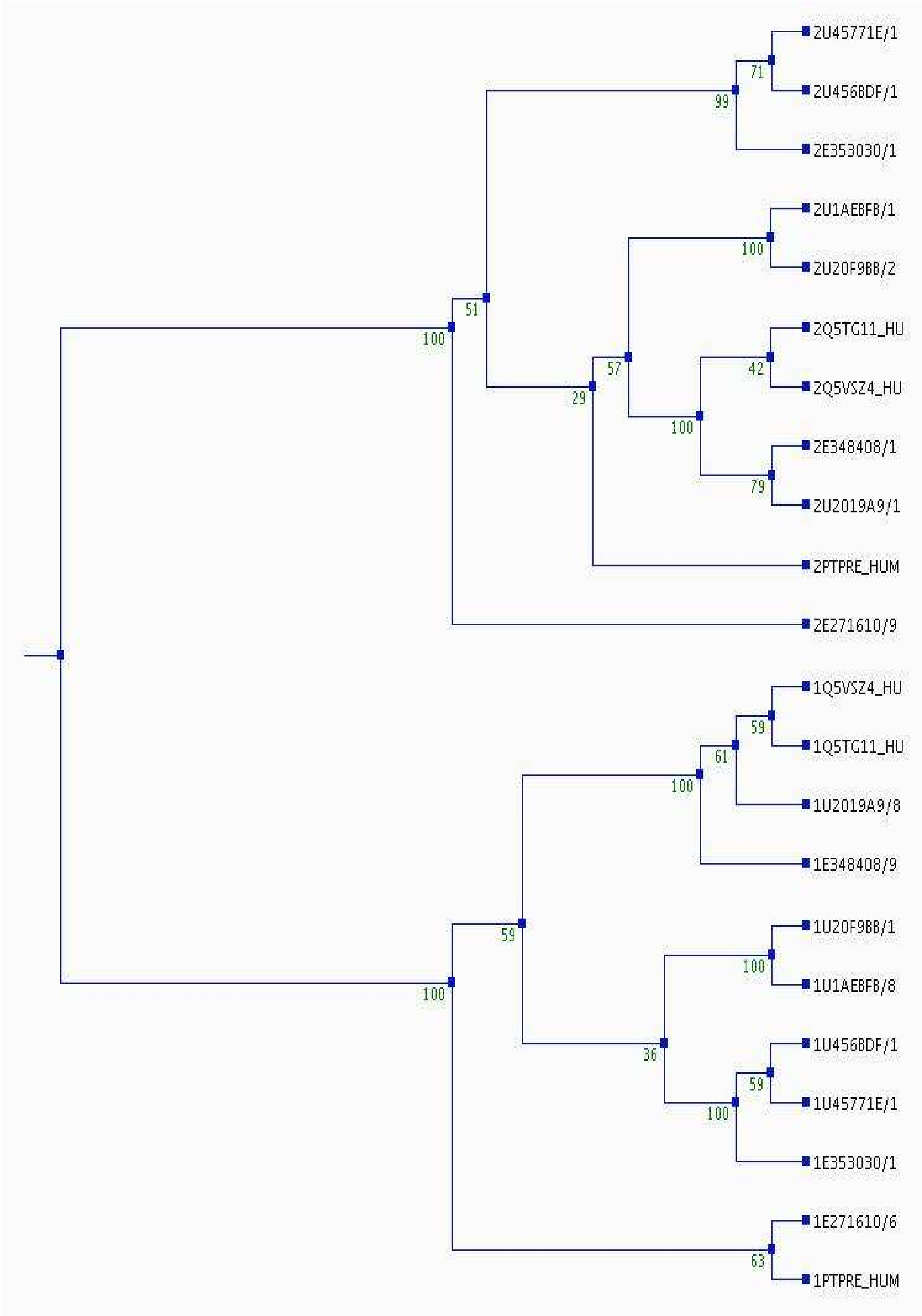

Figure 2: Bootstrap tree for the receptor tyrosine phosphatase data set.

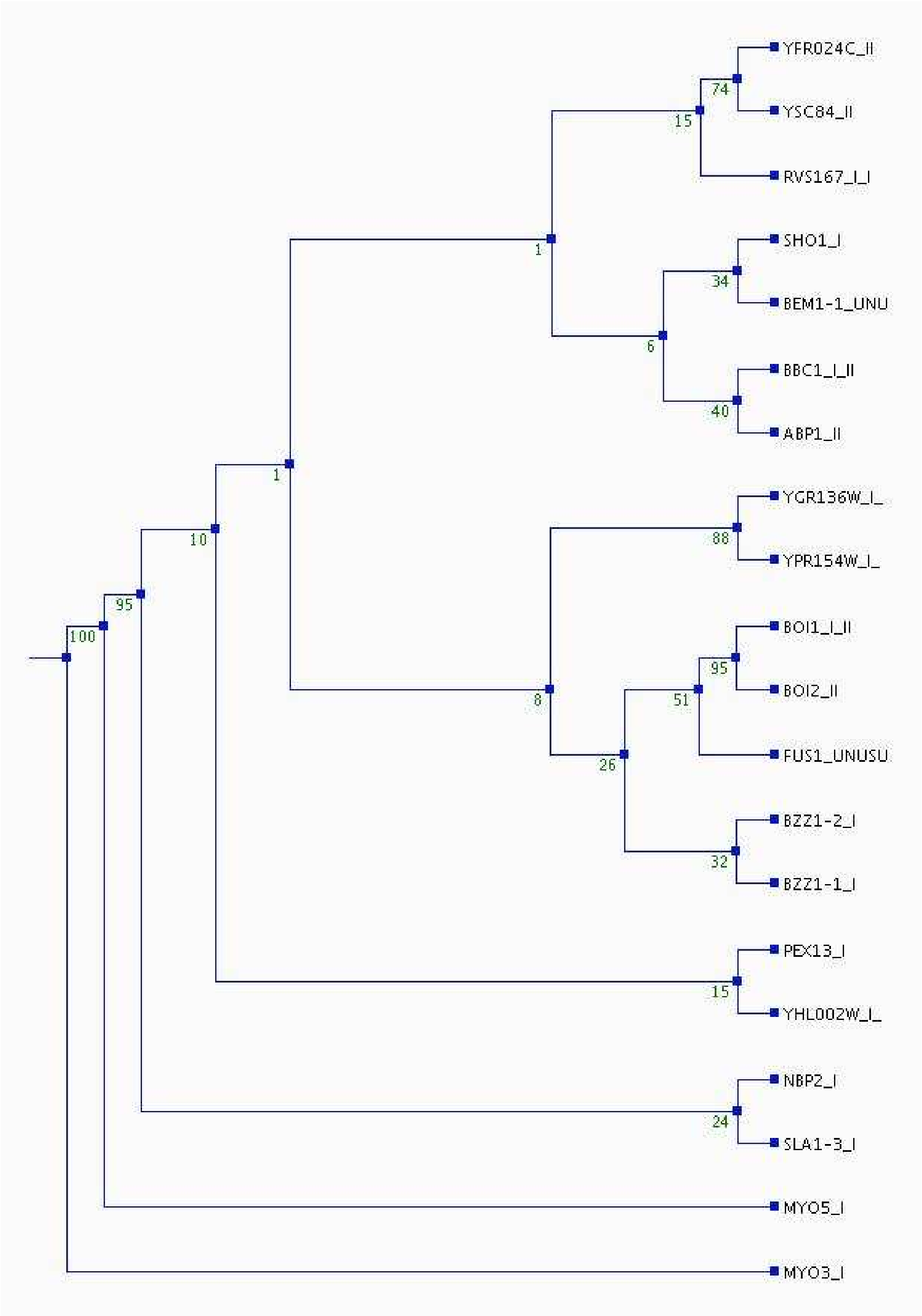

Figure 3: Bootstrap tree for the SH3 data set.

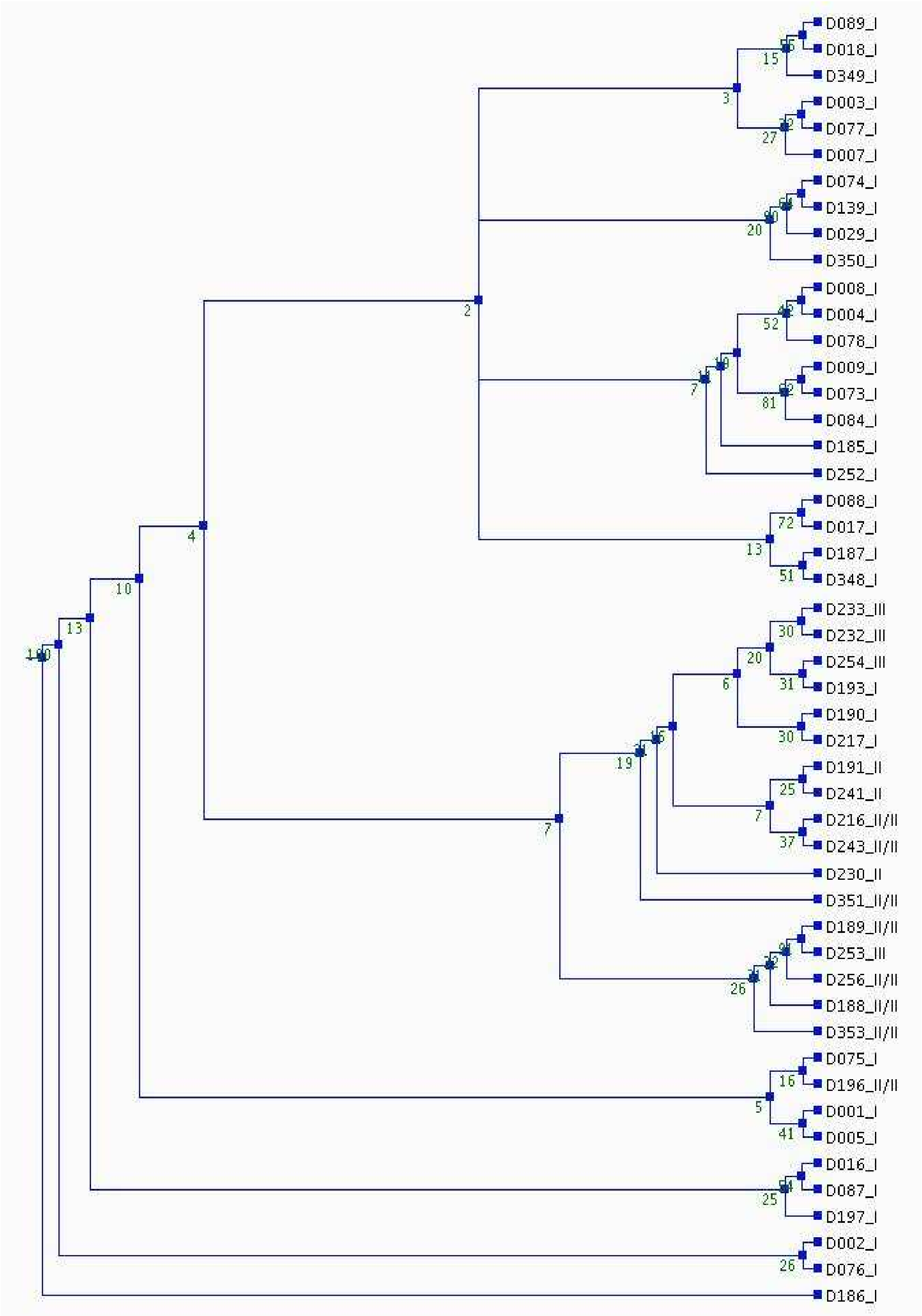

Figure 4: Bootstrap tree for the WW data set.
